# Supplementary material for: Conditional chemoconnectomics (cCCTomics) as a strategy for efficient and conditional targeting of chemical transmission
Source: eLife. 2024 Apr 30;12:RP91927. doi: 10.7554/eLife.91927 (PMC11060718; doi:10.7554/eLife.91927)
Supplement: Figure 3—source data 3. [file elife-91927-fig3-data3.zip › Figure 3 source data3 part 2.zip!/Figure 3-Source data 3-part2/1060-9-CRISPResso_Report_q0_g4o36.zip!/CRISPResso_on_1060_11/1a.Read_barplot.pdf]

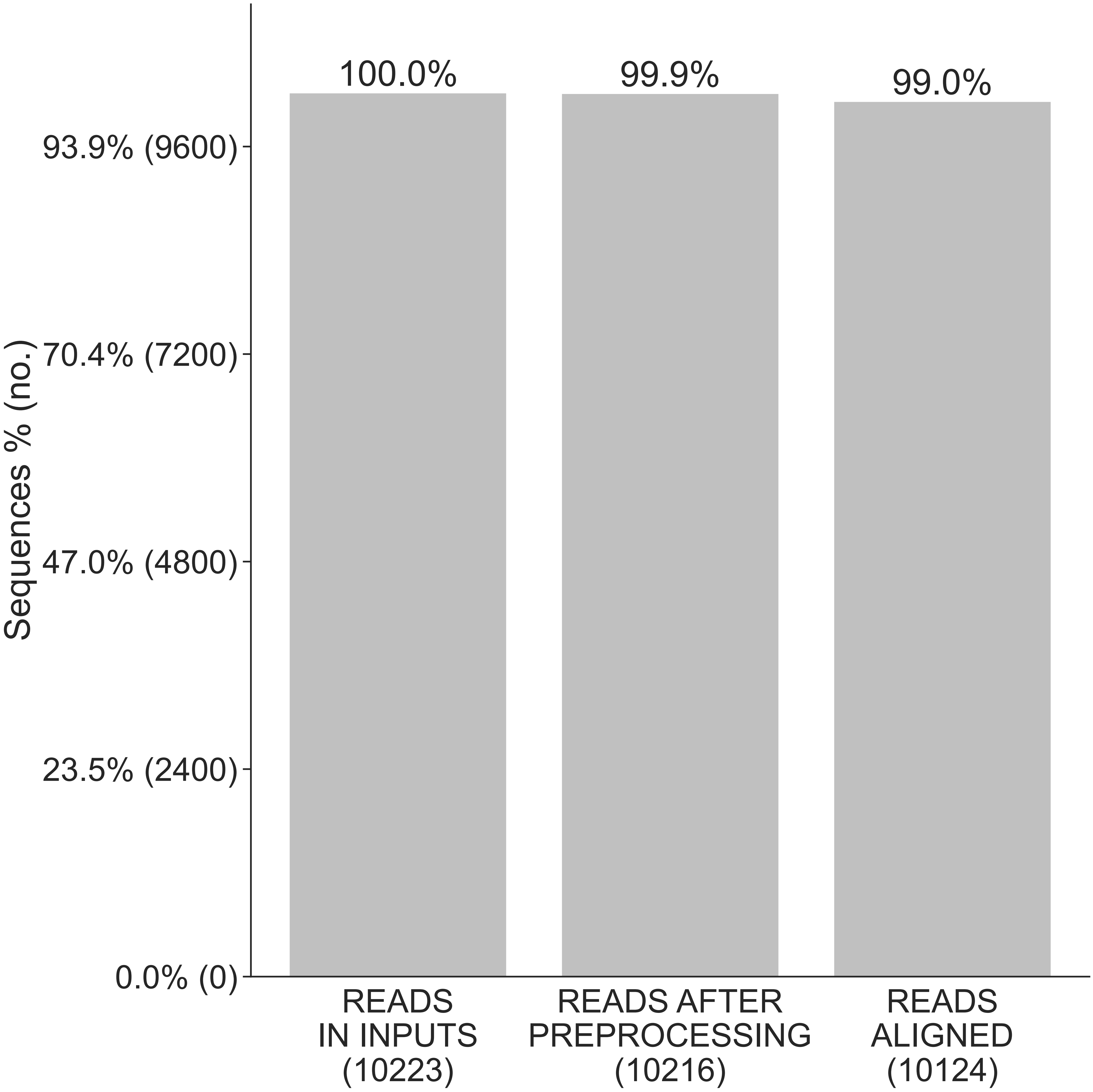
Sequences % (no.)
0.0% (0)
23.5% (2400)
47.0% (4800)
70.4% (7200)
93.9% (9600)
100.0%
99.9%
99.0%
READS IN INPUTS (10223)
READS AFTER PREPROCESSING (10216)
READS ALIGNED (10124)
